# Cooperative Binding of Transcription Factors Promotes Bimodal Gene Expression Response

**DOI:** 10.1371/journal.pone.0044812

**Published:** 2012-09-12

**Authors:** Pablo S. Gutierrez, Diana Monteoliva, Luis Diambra

**Affiliations:** 1 Centro Regional de Estudios Genmicos, Universidad Nacional de La Plata, Florencio Varela, Buenos Aires, Argentina; 2 Instituto de Física, Universidad Nacional de La Plata, La Plata, Buenos Aires, Argentina; Queen’s University Belfast, United Kingdom

## Abstract

In the present work we extend and analyze the scope of our recently proposed stochastic model for transcriptional regulation, which considers an arbitrarily complex *cis*-regulatory system using only elementary reactions. Previously, we determined the role of cooperativity on the intrinsic fluctuations of gene expression for activating transcriptional switches, by means of master equation formalism and computer simulation. This model allowed us to distinguish between two cooperative binding mechanisms and, even though the mean expression levels were not affected differently by the acting mechanism, we showed that the associated fluctuations were different. In the present generalized model we include other regulatory functions in addition to those associated to an activator switch. Namely, we introduce repressive regulatory functions and two theoretical mechanisms that account for the biphasic response that some *cis*-regulatory systems show to the transcription factor concentration. We have also extended our previous master equation formalism in order to include protein production by stochastic translation of mRNA. Furthermore, we examine the graded/binary scenarios in the context of the interaction energy between transcription factors. In this sense, this is the first report to show that the cooperative binding of transcription factors to DNA promotes the “all-or-none” phenomenon observed in eukaryotic systems. In addition, we confirm that gene expression fluctuation levels associated with one of two cooperative binding mechanism never exceed the fluctuation levels of the other.

## Introduction

At the transcriptional level gene expression is mainly controlled by the transcription factor (TF) proteins that bind specifically to regulatory binding sites on the DNA [1], [2]. TFs influence transcription rates by interacting with other components of the core transcriptional apparatus, including RNA polymerase. Due to the fact that TFs bind to DNA regulatory sites in a stochastic fashion, the transition between states of the *cis*-regulatory systems (CRS) is a stochastic process. Since the number of TF molecules and the number of regulatory sites are too small, the deterministic assumptions, which are valid in macroscopic systems, fail to describe a mesoscopic system such as this [3]. Therefore, due to the fundamentally random nature of chemical reactions, trajectories of individual cells are noisy and do not follow a smooth deterministic course. It is known that the gene expression response of an individual cell to a regulatory signal may be graded or binary [4]–[6]. In the graded response, the output varies smoothly with the input stimulus, whereas in the binary response, also termed the “all-or-none” phenomenon, gene expression response mainly occurs at either low or high levels. In the latter case, the resulting heterogeneous response of an ensemble of cells leads to a bimodal distribution of the protein level. This is a mechanism that can contribute to phenotypic diversity in genetically identical cell populations and is critical for increasing population survival in a fluctuating environment [7]. The bimodal response of gene regulatory networks can arise from closed loops (e.g., a two-gene system whose proteins mutually repress their transcriptional activity) or a single gene (where the gene expression product induces its own expression). These systems present bistability and have been reported previously [8]–[11]. Additionally, the “all-or-none” gene expression response has also been experimentally observed in some eukaryotic systems that do not involve bistability [4], [5], [12]–[14], where gene expression often occurs in stochastic bursts. This suggests that the binary responses observed in inducible gene expression could be explained by fluctuations in the binding of TFs to DNA [6], [15].

Contrariwise to prokaryotic RNA polymerases, eukaryotic polymerases require the prior assembly of general TFs at the typical eukaryotic promoter [16], [17]. These factors assemble in a particular order, beginning with the binding of TFIID to the TATA box. The ordered assembly provides several stages at which the initiation of transcription can be regulated [18], [19]. Thus, eukaryotic TFs can either facilitate or hinder the assembly of the transcriptional complex. Consequently, it is of paramount importance to contemplate the potential diversity of the CRS architecture and functionality when considering the various known mechanisms by which proteins and DNA interact [20]. However, most of the existing stochastic models for gene regulation are based on transitions between two CRS states (active and inactive) [21]–[26]. Despite their simplicity, these models extract valuable information about gene expression fluctuation. For example, they have illustrated that graded responses arise from fast chemical kinetics, whereas slow kinetics lead to a binary output [3]. Nevertheless, simple models may not be suitable for studying the role of different mechanisms that participate in complex transcriptional regulation processes.

Recently we proposed a mathematical model for transcriptional regulation in cooperative activator switches, which considers a CRS with several regulatory binding sites for a single kind of activator molecule [27]. In this study, by means of the master equation approach, we derived analytical expressions for the first two moments of the steady-state probability distribution for mRNAs and identified two cooperative binding mechanisms [27]: (i) the recruitment mechanism (RM) where the interaction between TFs increases the probability of binding another TF to DNA; (ii) the stabilization mechanism (SM), where the interaction between TFs decreases the unbinding rate of TFs from DNA. These mechanisms affect the fluctuation level in different ways, but not the mean response [27]. In the present paper, we demonstrated what we previously suggested by examination of some regions of the parameters space [27]: that the stabilization cooperative binding mechanism always presents a level of fluctuation greater than or equal to the recruitment mechanism.

Furthermore, in this paper we incorporate two novel generalizations to our previous model: (i) the capacity to understand cooperative mechanisms for repressor or biphasic switches, by considering that bound TFs can repress transcriptional complex formation and modulate transcriptional initiation in different ways [28]; (ii) the inclusion of analytical expressions for the first two moments of the steady-state probability distribution for proteins, enabling contrastation of theoretical and experimental data. In addition, this is the first study to show that cooperative binding plays an important part in determining the transition from graded to binary responses. In this sense, we establish the parameter space regions where each cooperative binding mechanism presents a graded or a binary response. Thus, our findings show that, as well as slow kinetics [3], cooperativity plays a key role in determining the transition from graded to binary responses.

## Methods

### A General Framework for Complex CRS Modeling

Here we present a framework for study models with many states and an arbitrary number of transitions between the different states. This extension of our previous model [27] includes the stochastic production of proteins.

**Figure 1:**
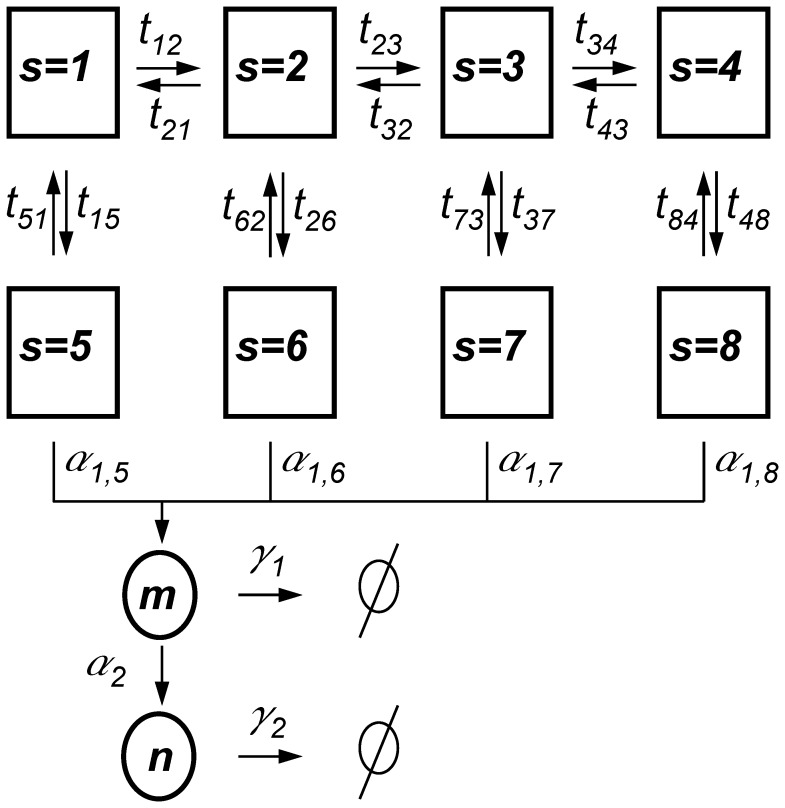
Schematic diagram of a complex *cis*-regulatory system. The model illustrated in this diagram includes eight states that are denoted by 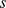. The allowed transitions between CRS states are indicated by arrows. A transition from state 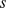 to state 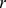 can occur with probability 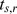. States with 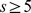 have been associated with no null rates of mRNA production 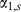, which depends on 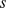. Each mRNA generates proteins at a constant rate 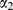. Both mRNAs and proteins are linearly degraded at rates 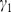 and 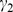, respectively.

In principle, the CRS states can represent nucleosome organization, DNA loops, TFs bound or unbound to regulatory sites, RNA polymerase binding, etc. Figure 1 depicts a particular outline for this type of complex model, considering eight possibles states, denoted by 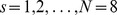, and fourteen allowed transitions. In general, the CRS can make transitions from a given state 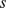 to state 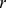 with probability 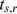. Some CRS states are able to synthesize mRNAs at a state-dependent rate, 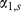. Each mRNA generates proteins, at a constant rate 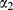. Thus the state of the system is specified by three stochastic variables: the chemical state of the CRS 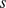, the number of mRNAs 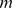 and the number of proteins 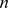. 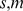 and 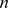 are integers, where 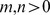 and 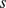 is 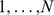. The model also assumes both mRNAs and proteins are degraded at rates 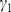 and 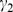, respectively.

Since our model assumes transcriptional regulation as a stochastic process, the theory of stochastic processes is required to analyze the resulting heterogeneous response of an ensemble of cells to a particular signal. Like other authors [21]–[23], [26], [27], [29], we used the master equation approach to study the average gene expression response in the steady state. We can write the probability of finding, at any given time 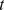, the system in the state 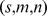 as a vector 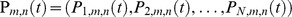. The time evolution for this probability is governed by the following master equation:
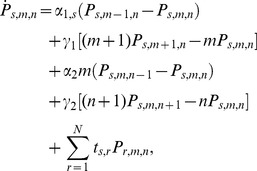
(1)where 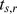 is the transition probability per time unit from state 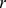 to state 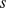. The last term on the right-hand side of Eq. (1) describes the CRS dynamics, while the others correspond to the production and degradation of mRNAs and proteins. Unlike the master equation for the previous model [27], Eq. (1) has a new random variable 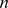 corresponding to proteins and two new terms associated with their production and degradation.

### The Steady-state Solution

A time-dependent solution of Eq. (1) is very difficult to obtain even in simpler models. Nevertheless, we are mainly interested in the steady-state solution for mRNA and protein mean levels and their fluctuations. By elaborating on the approach developed in [27], we were able to compute the first two moments of these quantities. The mean levels are measured through the first moment of the number of mRNAs 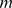 and proteins 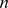,
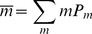
(2)

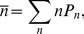
(3)where 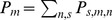 is the marginal probability of the system to have produced 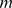 mRNAs, regardless of both the CRS state and the number of proteins for that state, while 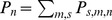 is the marginal probability of the system to have 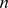 proteins, regardless of both the CRS state and the number of mRNAs for that state. The fluctuations are measured through the corresponding variances, related to the second moments,
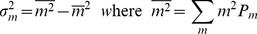
(4)

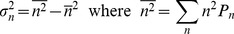
(5)


The summation limits were suppressed for the sake of readability. From now on, every sum over mRNAs or proteins will run from 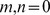 to 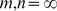, while the sum over CRS states will be from 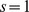 to 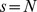.

Following [27], the moments of 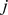
th order can be written in terms of their associated partial moments. Note that the partial moments of order zero are the marginal probabilities for the operator to be in state 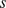 at time 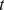, 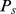, regardless of the number of mRNAs or proteins present at this time, i.e., 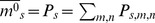,
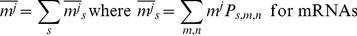
(6)

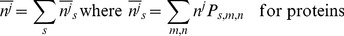
(7)From Eq. (1) we can derive a set of ordinary differential equations for the time evolution of the partial moments for any 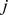. As there is no feedback, the equations for the partial moments factorize into independent sets of linear equations, which can easily be solved. For 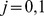, and 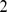 they are 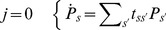
(8)

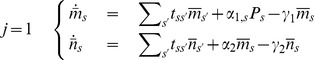
(9)

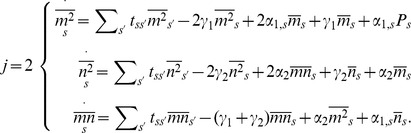
(10)


From these we can readily find first-order differential equations governing the time evolution of the first moments and variances
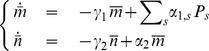
(11)

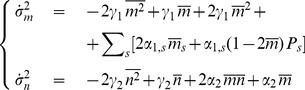
(12)
Equations (11) immediately reduce, in their steady states, to.
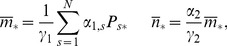
(13)where the above 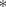 denotes the steady-state solution for the random variable. The steady-state solution for the probability vector 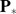 corresponds to the normalized eigenvector related to the zero eigenvalue of the CRS transition matrix, 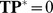. From Eqs (12) for the steady-state variances we find 
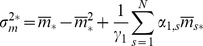
(14)

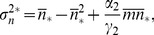
(15)where from the last differential equation for the 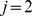 partial moments, Eqs (10), the second order moment in its steady state 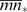 can be related to the steady-state first-order partial moments of 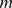 and 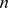 by
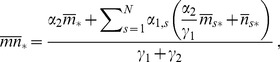
(16)and where 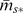 and 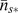 are determined as the solution of the linear equations 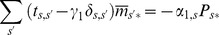
(17)

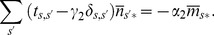
(18)


Expressions (13)–(18) are general in the sense that they are valid for any CRS, whatever the details of their dynamics. The expressions for mRNA have been previously reported in [27]. Here we incorporate the expressions of mean and standard deviation for proteins, which will allow contrasting models with experiments as many times as experimentalists assess protein levels. The expression for protein fluctuations predicted here does not differ only in an offset from the mRNA fluctuation, as was found in a previous study that considers a many-state CRS [29]. This model assumes that each mRNA generates a burst of proteins, whose size is geometrically distributed. Indeed, our resulting expressions for steady-state fluctuations of mRNAs and proteins, expressed in the form of normalized variance, conform to the general equation described previously by Paulsson [24], but with a more complicated term for the activation-inactivation transitions. Thus, our results expand upon previous studies that were either limited to the modeling of promoter state transitions as a two-state on/off switch [3], [21], [24], [26] or which excluded translation when more than two promoter states were modeled [27], [30].

### Modeling Genetic Switches

The expressions of the previous subsection are independent of the specific form of the CRS transition matrix 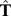 and of the number of states 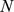. In this section, we will specify the CRS states and the form of the transition matrix 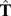 associated with a particular CRS that is suitable for modeling the transcriptional regulation of switches. For the sake of simplicity, we will consider only eight states (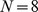) as sketched in Fig. 1. In order to study the cooperative regulation our model includes three regulatory binding sites for the same TF (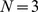), but the generalization to an arbitrary number of sites is straightforward. As in [27], the states 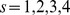 represent states with zero, one, two, and three binding sites occupied by TFs, respectively. The states 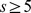 correspond to transcriptional preinitiation complex formation, where all components required for transcription are assembled in the CRS. For simplicity, we consider that TFs do not bind or unbind after the formation of the preinitiation complex; the allowed transitions between the CRS states are indicated by arrows in Fig. 1. Once the core transcriptional apparatus is formed, the synthesis of one mRNA copy begins at rate 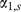. Each mRNA generates proteins at a constant rate 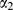. Our model also assumes that both mRNAs and proteins are linearly degraded at rates 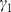 and 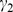, respectively. In the model we can distinguish four regulatory layers. Layer I corresponds to CRS dynamics of TF binding to/unbinding from DNA, layer II corresponds to preinitiation complex formation, layer III corresponds to mRNA production/degradation, while layer IV corresponds to protein production/degradation.

In order to obtain the explicit expressions of the steady-state solutions in terms of the parameters of the system, we need to specify the CRS transition matrix 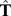. The TFs can bind to regulatory sites with a probability proportional to TF concentration 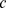, following the law of mass action for elementary reactions. Thus, the transition probabilities 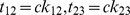 and 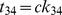, while transition rates 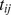 to and from other states of the operator are denoted simply as 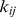. In this case the transition matrix 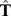 can be written as
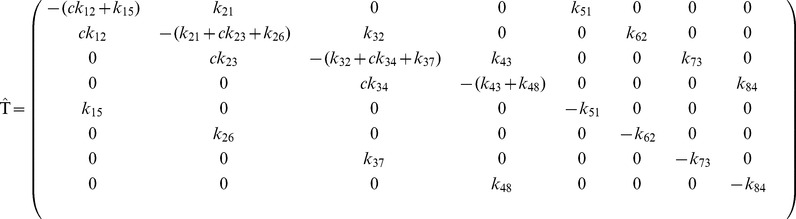
(19)whose associated steady-state solutions of the partial probabilities 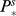
involved in Eq.(13) were calculated in [27]. The explicit expression for levels of mRNAs in the steady state is
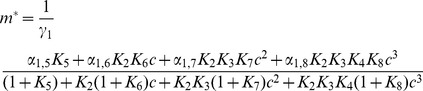
(20)where 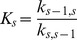 for 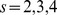 and 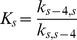 for 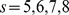. Closed expressions for the variances can also be obtained for 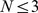 but are too long to be reported here. As in this paper we deal with steady states only, hereafter we write 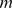 to denote 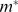.

The working hypothesis in our model is that TFs bound to DNA alter the probability of transcriptional complex formation. Consequently, states 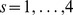 are characterized by different kinetics for the formation of the preinitiation complex. For simplicity, we consider that the sites are functionally identical. The last assumption implies that the model does not distinguish among states with the same number of TFs bound to the regulatory binding sites. Thus, in our model, the states of CRS are more related to the occupancy number rather than to the binding status of each site. This additional simplification reduces the number of states accessible to the CRS and allows us to explore the role of cooperative binding in the noise expression without considering a combinatorial number of states. In this model, with several states able to transcribe, it will be useful to define the transcriptional efficiencies 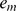 for each occupational number 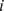 as the rate of mRNA production when there are 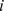 TFs bound to DNA, i.e., 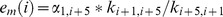 for 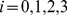.

As in the model the regulatory sites are assumed to be functionally identical, we can introduce a relationship between TF binding/unbinding when there is no interaction between the TFs. Thus, if the probability per time unit that a single TF molecule binds to a regulatory site is 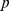, we have 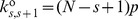, with 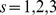, and ° indicates that there is no interaction between TFs. Similarly, unbinding rates are given by 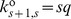, where 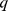 is the probability per time unit that a single TF molecule unbinds from an occupied site.

A further relationship in layer I can be obtained from the principle of detailed balance, which establishes a relationship between the kinetics and the thermodynamic properties of the system [31]. Thus, we will assume that the probability for a TF molecule to bind to a given regulatory site arises from: (i) the free energy of binding a TF to the specific site 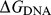, (ii) the free energy of interaction between TF molecules bound to adjacent sites 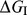. Thus, when there is no TF interaction, we have
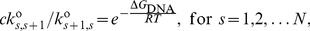
(21)where 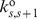 represents the transition rate from state 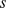 to state 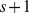 when there is no interaction between TFs (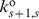 represents the rate of reverse transition) and where 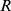 is the gas constant and 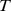 is the absolute temperature. In general, the TF molecules interact with each other, i.e., 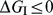. If we now assume that each new bound TF interacts with all TFs already bound to the DNA sites, and furthermore, that this energy is the same for all of them, we have
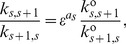
(22)where 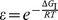 represents the intensity of the interaction between TFs and 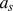 represents the number of interactions, which, because of our assumption, will be 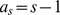 with 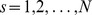.

Relationship (22) leaves an extra degree of freedom, because the interaction between TFs can increase the binding rate 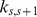, increasing the ability for the recruitment of new TF for DNA binding, or it can diminish the unbinding rate 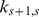, increasing the stability of the TF bound to DNA. The first case was denoted as the RM, while the second case was denoted as the SM [27]. In order to understand the effect of these cooperativity binding mechanisms on the regulatory response and their associated fluctuations, we will first consider these mechanisms separately. Thus, using relation (22) and the relations for binding/unbinding rates, we obtain
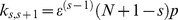


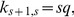
(23)for the first mechanism, while for the second mechanism we have



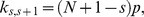


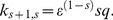
(24)Additionally to the two cooperativity binding mechanisms mentioned above, introduced for the first time in [27], we will here consider the case where both mechanisms are acting simultaneously. In this case, we can write the free energy of interaction as 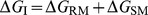, where 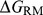 corresponds to the free energy that increases the ability for new TF recruitment for DNA, while 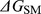 corresponds to the portion of the free energy that diminishes the unbinding rates 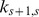. Thus, in this more general scenario, we can write the kinetic constants of layer I as



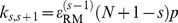


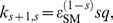
(25)where 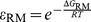, and 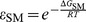, noting that 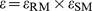. These thermodynamic relationships allow us to write the kinetic parameters of layer I in terms of three parameter 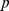, 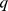 and 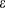.

In the next section we will study the transcriptional response of CRS when the TF concentration 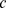 is increased. The mean response can be characterized by three parameters: (i) the saturation value (known as 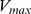), which is defined as 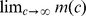; (ii) the half-maximum concentration (denoted here by 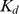), which is defined as the concentration 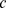 at which 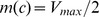 (i.e., 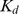 is a root of the polynomial of degree 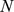); (iii) the steepness 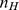, which is defined as
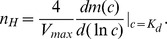
When these definitions are applied to a Hill function 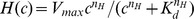, one can determine the three parameters (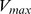,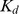,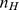) related to 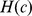. The above definition allows us to characterize the sigmoidal response given by Eq. (20) analytically, avoiding a nonlinear fitting procedure. Additionally, we characterize the fluctuation around the mean transcript number by the value of standard deviation, given by Eqs. (12) and (15), at 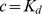, which is denoted by 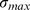.

## Results

### Activator, Repressor and Biphasic Switches

In [27] we reported two different cooperative binding mechanisms for activator switches. Here we expand the proposed model to include different types of switches by appropriately setting kinetic rates in layer II and/or in layer III. For example, a repressor switch is obtained if the transcriptional efficiencies 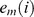 decrease monotonically with the occupancy number 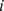. This means that, in the example of Fig. 1, 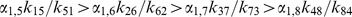. On the other hand, if there is a nonmonotonic dependence of 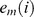 with 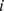 we are dealing with a switch with biphasic response to the TF.

The kinetic parameter values used here are listed in Table 1. For typical experimental conditions, 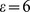 corresponds to 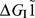 kcal/mol. This value is similar to the interaction energy between two 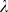-repressor molecules [32] and a bit higher than the free energy associated with the cooperative binding of E2 proteins (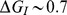 kcal/mol.) [33]. The binding and unbinding rates of TFs are consistent with the measured values for the *lac* repressor [34], and for E2 [35], when the TF concentration is given in nM and 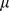
M, respectively. Other parameters are assigned plausible but arbitrary values, due to the absence of kinetic information with regard to the other state transitions.

**Figure 2:**
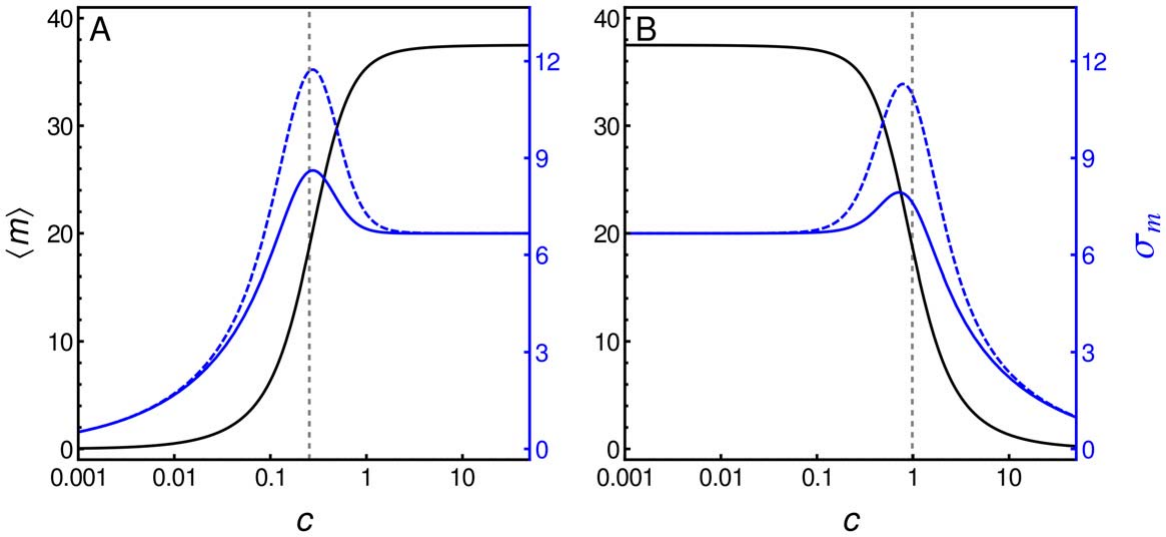
Activator and repressor switches. Average number of mRNA copies 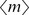 (black lines) and the associated standard deviations 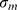 (blue lines), as a function of TF concentration 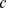 in steady state for two different types of switches: activator switch (A), repressor switch (B). The standard deviations corresponding to the recruitment mechanism are indicated with solid lines, while dashed lines correspond to the stabilization mechanism. Vertical gray dashed lines are at 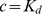. See Table 1 for parameter values.

Here we consider activator and repressor switches where the kinetic rates for preinitiation complex formation increase or decrease linearly with the occupancy number, respectively. Figure 2 depicts the average number of mRNA copies 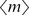 and the associated standard deviations 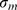 as a function of the transcription factor concentration 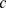, obtained analytically for both cooperative binding mechanisms for activator (A) and repressor (B) switches. Both cooperative binding mechanisms present the same 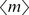 response. The behavior of the mean and the standard deviation related to the repressor is very similar to the activator response but as expected, with the 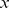-axis reflected. The regulatory functions of examples 2A and 2B present steepness of 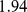 and 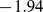, respectively, and the same saturation value. However, activator and repressor differ in the 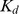 value and in the noise level. For the kinetic parameters used in this case the repressor (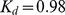) is less sensitive than the activator (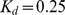). We also observe that the peak of 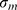 associated with the repressor is slightly smaller than that associated with the activator.

**Table 1:** Kinetic parameters.

TF binding and unbinding (Layer I)		Preinitiation complex formation (Layer II)			
			activator	repressor	biphasic
*p*	0.25	*k*_15_	0.00	1.50	0.01
*q*	0.75	*k*_26_	0.50	1.00	2.00
*ϵ*	6.00	*k*_37_	1.00	0.50	2.00
		*k*_48_	1.50	0.00	0.01
		*k_s_*_,*s*−4_	0.50	0.50	0.50
Production and degradation rates					
mRNA (Layer III)		*α*_1,s_	1.50	γ_1_	0.03

Kinetic parameter values for figures. The time unit is min and the concentration is an arbitrary unit.

**Figure 3:**
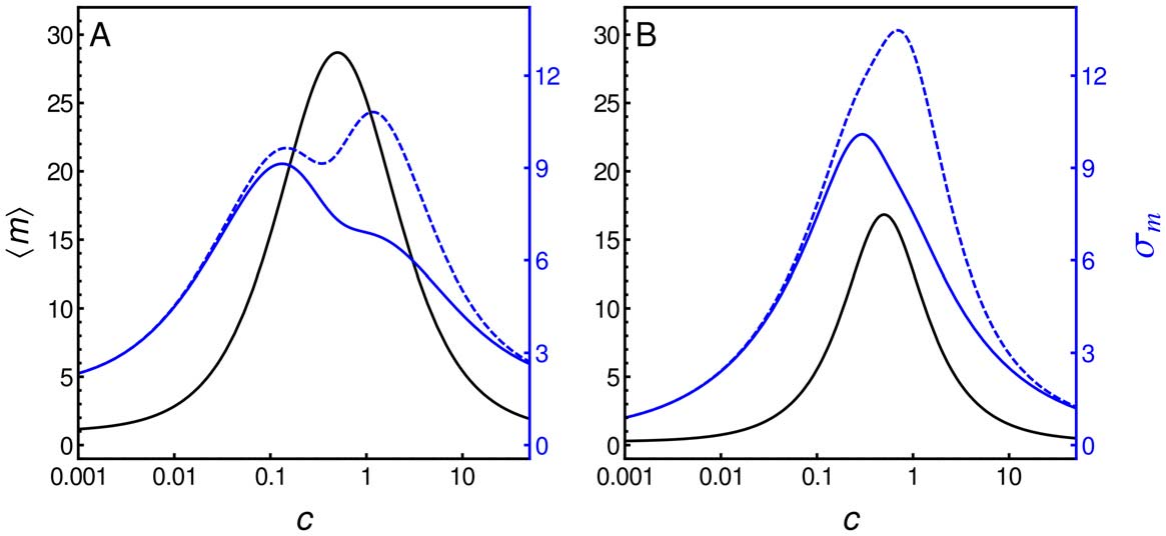
Biphasic switches. Average number of mRNA copies 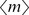 (black lines) and the associated standard deviations 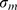 (blue lines) as a function of TF concentration 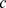 in steady state for two biphasic switches: the biphasic response originated by layer II modulation (A) and by layer III modulation (B). The standard deviations corresponding to the recruitment mechanism are indicated with solid lines, while dashed lines correspond to the stabilization mechanism. For the last mechanism 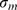 has two peaks only in panel A. Parameters for panel A are listed in Table 1. Panel B parameters are 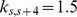 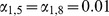 and 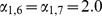, while the rest of the parameters correspond to those in Table 1.

**Figure 4:**
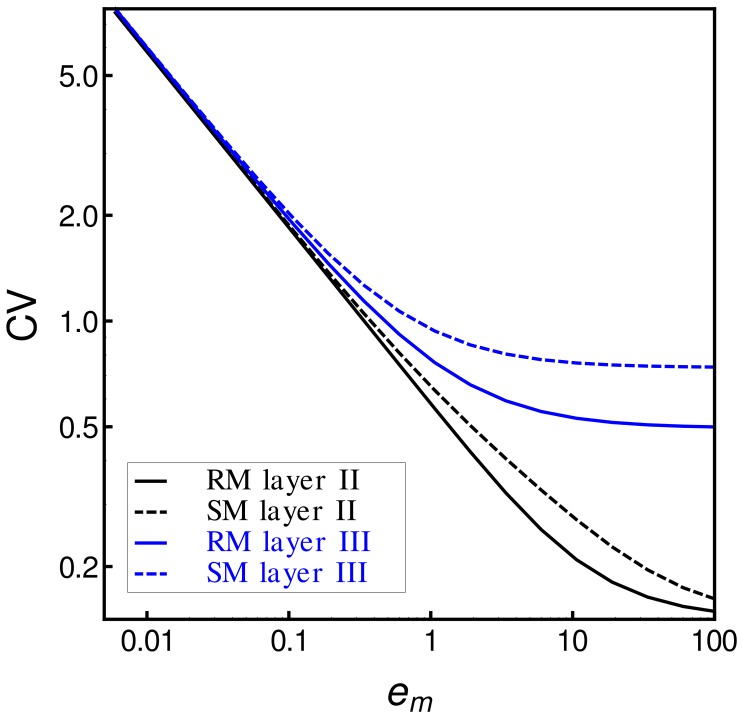
Modulation of layer III generates more fluctuation than modulation of layer II. Coefficient of variation (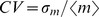) associated with the above-mentioned biphasic switches as a function of the overall transcription rate 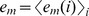. Black lines correspond to the 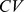 from biphasic switches with layer II modulation, while blue lines correspond to biphasic switches with layer III modulation. Solid lines correspond to RM, while dashed lines correspond to SM.

Figure 3 illustrates two examples of biphasic switches when the transcriptional efficiency 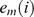 does not depend monotonically on the occupancy number 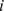. In Fig. 3A the modulation of the transcriptional efficiency occurs in layer II, while in Fig. 3B the biphasic response to the TF is obtained by the modulation of the rates 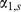 (i.e., layer III). In both cases mean responses are biphasic. Again the mean response does not depend on the cooperative binding mechanism which is acting. In Fig. 3A the fluctuation level, estimated by the standard deviation 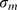 associated with the SM has peaks near the two values of the concentrations where the response is half the maximum, while in the RM case 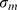 presents only one peak. The fluctuation level around the second half-maximum concentration depends strongly on the acting cooperative binding mechanism. In order to observe the effect of the second type of transcriptional efficiency modulation, we keep the same overall transcription rates by setting 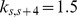 for all 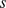, 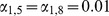, and 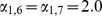. In this case, depicted in Fig. 3B, the mean response decreases and the standard deviation has only one peak with higher amplitude than in the previous case in which the modulation is acting over the kinetic rates related to layer II. We also compare the fluctuation levels associated with these two types of biphasic switches for different transcriptional efficiencies. In this sense, we compute the coefficient of variation 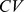, as noise measurement (defined as 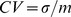) at the TF concentration 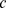 where 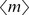 reaches the maximum, as a function of the overall transcriptional efficiency 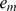. In the case of a biphasic switch with modulation of the layer II kinetics, different values of 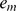 are obtained by increasing the rates 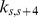 and keeping 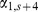 constant. For a biphasic switch originated by the modulation of layer III kinetics, this is done by increasing the rates 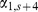 keeping the kinetic rates 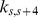 constant. When comparing the respective cooperative binding mechanisms at different transcriptional efficiencies (Fig. 4), we found that the biphasic switch with the latter modulation is always noisier than that where the biphasic response occurs due to the kinetics of layer II.

**Figure 5:**
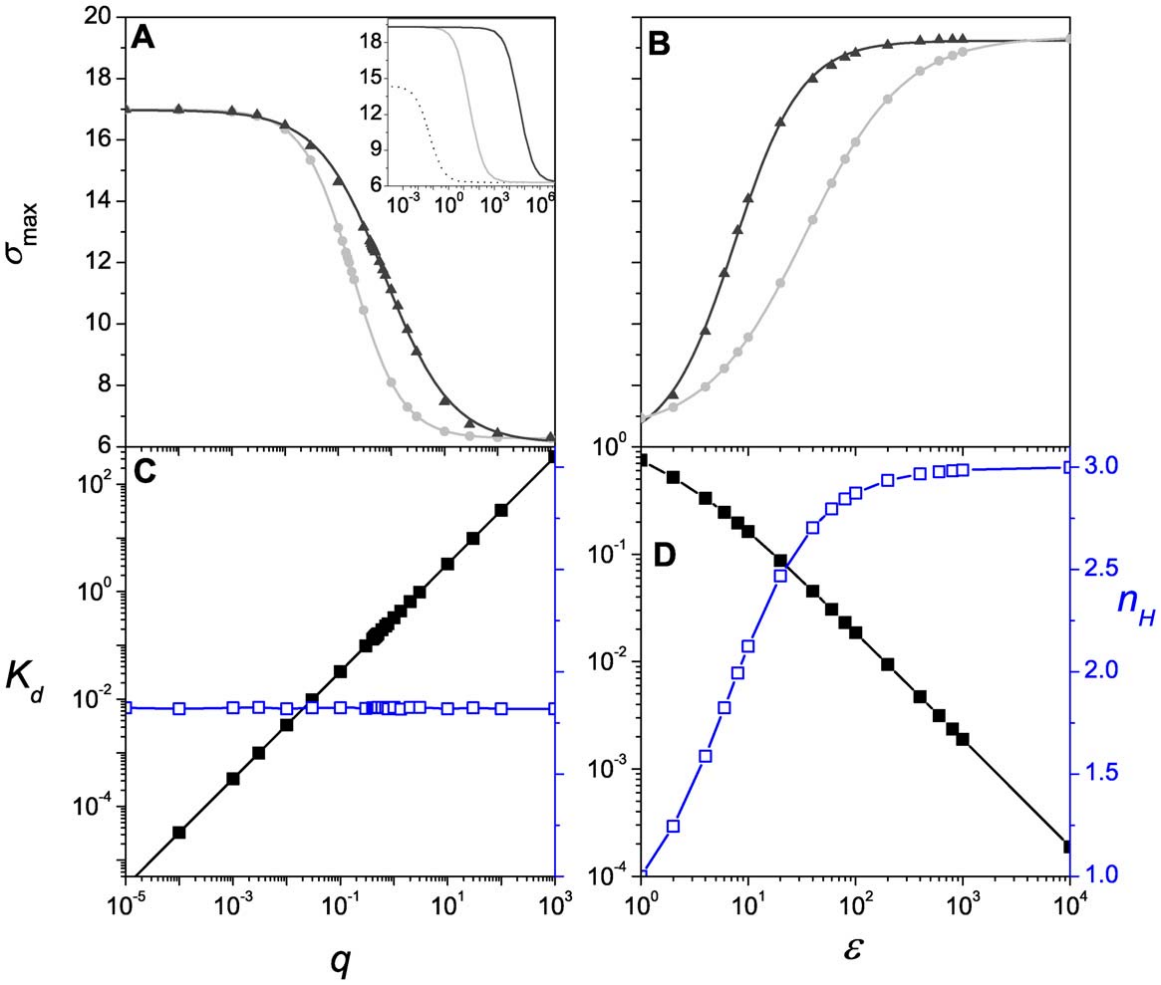
Activator response. (A) 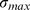 as a function of the unbinding rate 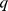 for the RM (gray) and the SM (black). Inset: 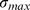 as a function of 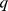 for the RM (gray) and the SM (black) obtained with 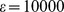. The dotted line depicts the noncooperative case (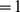). (B) 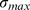 as a function of the cooperativity parameter 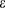 for the RM (gray) and the SM (black). (C) The dissociation constant 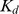 (black) and the steepness 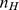 (blue) as a function of the unbinding rate 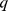. (D) The dissociation constant 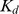 (black) and the steepness 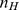 (blue) as a function of the cooperativity parameter 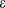. Parameters are the same as in Fig. 2A except for the varying parameter in each case. Lines correspond to analytic solutions and symbols to simulations.

**Figure 6:**
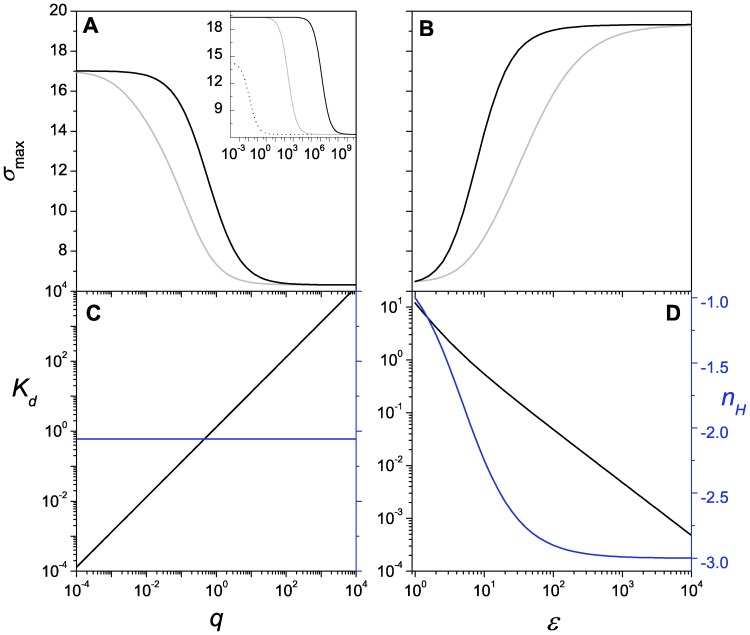
Repressor response. (A) 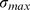 as a function of the unbinding rate 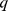 for the RM (gray) and the SM (black). Inset: 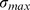 as a function of 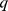 for the RM (gray) and the SM (black) obtained with 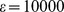. The dotted line depicts the noncooperative case (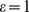) (B) 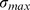 as a function of the cooperativity parameter 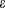 for the RM (gray) and the SM (black). (C) The dissociation constant 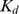 (black) and the steepness 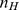 (blue) as a function of the unbinding rate 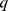. (D) The dissociation constant 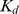 (black) and the steepness 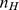 (blue) as a function of the cooperativity parameter 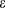. Parameters are the same as in Fig. 2B except for the varying parameter in each case.

In order to study how the response of CRS (i.e., the mean and the fluctuations of the mRNA level) depends on the cooperativity parameter 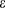 and on the unbinding rate 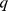, we computed three parameters to characterize the mean response and one to characterize the fluctuation around the mean (see subsection Modeling genetic switches). The binding rate 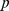 only affects the dissociation constant 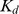 as reported in [27]. Figure 5 illustrates the activator response behavior of 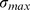, i.e. 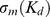, as a function of the unbinding rate 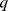 (Fig. 5A) and as a function of 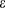 (Fig. 5B). In this case it is observed that 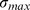 corresponding to the RM does not exceed that associated with the SM. 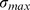 decreases sigmoidally with 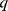. The saturation value at low 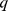 and the half-maximal 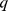-value increase with 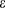 as can be seen in the inset of Fig. 5A. In fact, for the noncooperative case, 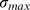 is lower than for the cooperative cases at low and intermediate values of 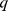, but equal at high values of 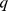. On the other hand, 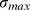 increases sigmoidally with 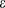 (Fig. 5B); again, 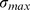 corresponding to the RM does not exceed that associated with the SM, but both saturate to the same value at high values of 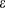. The curves of Fig. 5A and 5B were computed by evaluating the analytic expression for 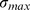, while the symbols were obtained by simulation using the Gillespie method [36]. Figure 5 also illustrates the behavior of the dissociation constant 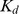 and the steepness 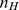 *vs.* the unbinding rate 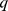 (panel C) and 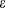 (panel D). As expected, the sensitivity decreases with the unbinding rate but increases with 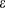. On the other hand, the steepness 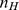 depends only on 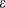 and not on 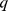 or 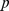 (data not shown). As expected, 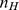 (blue line) increases with  and saturates at 3 at a high value of . A CRS with 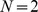 activation sites saturates at 2 (data not shown). Thus, in the limit 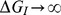, we can recover the Hill function from the expression of the mean response (Eq. 22). A similar behavior is observed for a repressor switch, Fig. 6, with the exception of the steepness 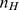 as a function of 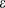 (Fig. 6D). In this case 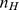 decreases with  and saturates at −3 at high interaction energy, as expected for a negative regulator. Further differences between Fig. 5 and Fig. 6 are the sensitivity and the fluctuation level. For these parameter values the repressor is less sensitive and noisier than the activator, as we noted in Fig. 2.

### Comparing the Cooperative Binding Mechanisms

Results presented up to this point suggest that the SM is associated with a level of noise greater than, or at least equal to, the RM. Now we are interested in determining whether this behavior is a general feature of these mechanisms or if a different scenario can be expected in some regions of the parameter space. In order to address this question we computed the difference between the variances of SM and RM. For the sake of simplicity we considered a switch with two binding sites. Such simplification is sufficient to consider the effects of the binding cooperative mechanisms and to reduce the number of CRS states to six allowing an analytical approach. That is, referring to Fig. 1, we set 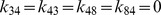, so as to keep only CRS states with 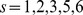 and 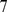. Noticing that the mean values 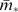 do not depend on the acting mechanism, such difference is given by
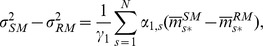
where 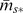 are solutions to Eq. (15), with the corresponding transition matrix 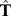, given by Eq. (19) for RM, and by Eq. (20) for SM. If we consider a switch with 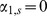 for 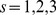 as discussed previously, then the difference between the variances of SM and RM can be written as



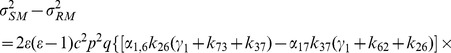


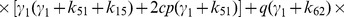


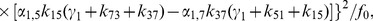
(26)where the denominator 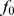 is a parameter dependent factor, which is the sum of positive terms and consequently it is always positive definite. The difference between the variances of SM and RM can be written as



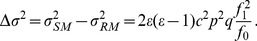
(27)The above expression for 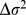 is positive for all the parameter space whenever 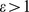, thus supporting the presumption that follows from our numerical results, i.e., that, for a switch, such as the one depicted in Fig. 1, but with two sites, the fluctuation level associated to the RM never exceeds the fluctuation level associated with the SM.

**Figure 7:**
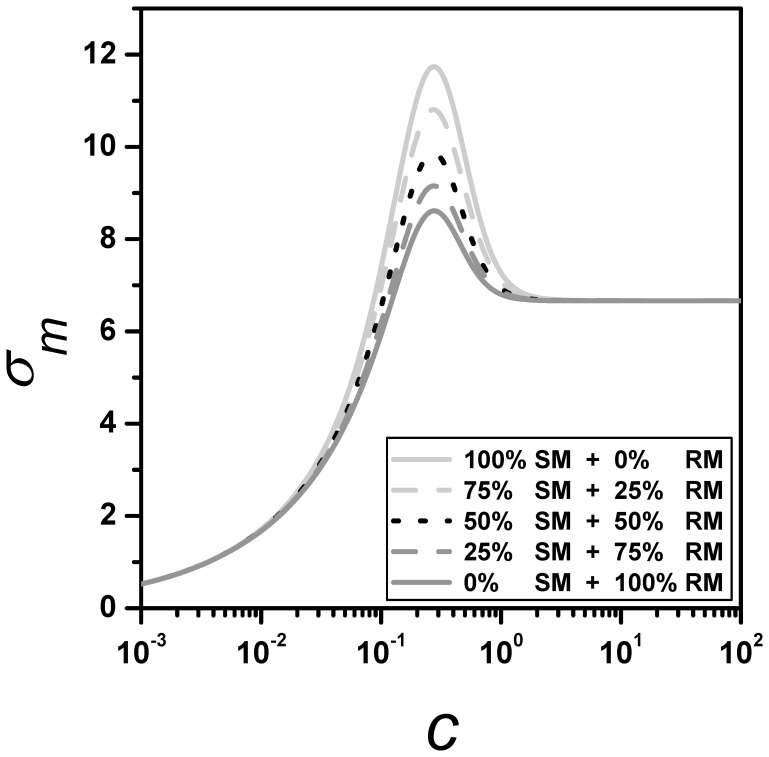
Effects of mixing cooperative mechanisms. Standard deviations of the number of mRNA copies, 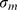, as a function of TF concentration 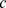. Curves correspond to an activator switch with the same parameters as in Fig. 2A but each fluctuation curve corresponds to different contributions from each mechanism. The solid light-gray line corresponds solely to the SM (i.e., 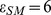 and 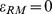), the dashed light-gray line corresponds to 75% and 25% contributions from SM and RM, respectively (i.e., 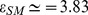 and 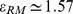), the dotted black line corresponds to equal contributions from each mechanism (i.e., 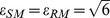), the dashed dark-gray line corresponds to 25% and 75% contributions from SM and RM, respectively (i.e., 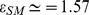 and 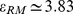), and the solid dark-gray line corresponds solely to the RM (i.e., 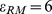 and 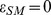).

In live organism, it is more plausible than these cooperative binding mechanisms act simultaneously rather than in an excluding manner, as illustrated for a clearer interpretation. In this context, we also consider some cases where both RM and SM are acting together. Figure 7 depicts the standard deviation 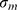 for an activator switch with the same  as Fig. 2, but each fluctuation curve corresponds to different contributions from each mechanism. The solid light-gray line corresponds solely to the SM, the dashed light-gray line corresponds to a contribution of 75% from SM and 25% from RM, the dotted black line corresponds to equal contributions from each mechanism, the dashed dark-gray line corresponds to a contribution of 25% from SM and 75% from RM, and the solid dark-gray line corresponds solely to the RM. From this plot, we can observe that fluctuations have a proportional dependence on the mechanism contributions.

### Graded and Binary Responses

**Figure 8:**
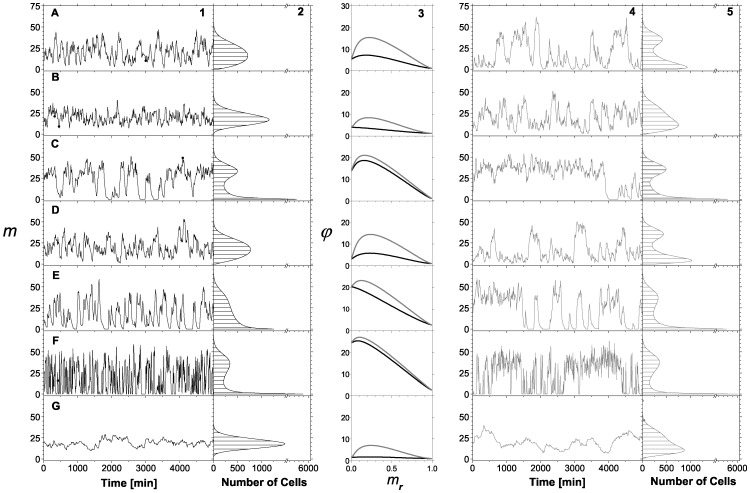
Comparing two cooperative mechanisms. Panels on columns 1, 2 and black lines of panels on column 3 correspond to the RM, where bound TFs increase the binding rate of new TFs to DNA. Panels on columns 4, 5 and gray lines of panels on column 3 correspond to SM, where the interaction between TFs decreases the unbinding rate of TFs from DNA. Time series of mRNA number generated from stochastic simulations (columns 1 and 4). Histograms that show the number of cells with a given number of mRNAs are also shown (columns 2 and 5). Strength noise 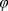 as a function of 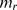 for both mechanisms (column 3). The above features were studied at different kinetic rates. Row A corresponds to the same kinetics shown in Fig. 2A. Rows B and C, all kinetic rates of layer I were multiplied by a factor of 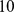 and 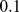, respectively. Rows D and E, all kinetic rates of layer II were multiplied by factors of 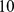 and 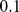, respectively. Rows F and G, all kinetic rates of layer III were multiplied by a factor of 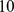 and 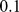, respectively. All simulations were performed using Table 1 parameter values, except for 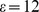 and 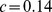.

While the influence of the different mechanisms in which cooperativity can affect the gene expression response is evident from Figs. 5 and 6, their effects are even more dramatic when the steady-state distribution function is studied. In Fig. 8 we compare the recruitment and stabilization mechanisms along several kinetic rates that render the same mean response function using the same kinetics as in the previous activator case (see Table 1), but with 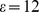. By multiplying all parameters related to a particular regulatory layer by a factor, we alter the fluctuation level, but not the mean response that depends on ratios rather than on individual kinetic rates. The first panel of row A, A1, is the time series of the mRNA number in one cell generated by stochastic simulations using the parameter values of the RM case. The associated histogram (panel A2) shows the number of times a cell shows a given number of mRNAs measured every 10 minutes over a population of 20000 cells. Panels A4 and A5 are the time series and histogram, respectively, of the mRNA number generated by stochastic simulations for the SM case. All time series and histograms in Fig. 8 were obtained using 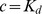. For comparison, in panel A3 of Fig. 8 we depict the noise strength 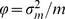 as a function of 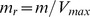, corresponding to the RM case and the SM case. Interestingly, the mechanism acting by stabilization which reduces the unbinding rates, presents a bimodal distribution (panel A5), while the cooperative recruitment mechanism with the same parameters 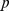, 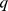 and 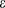, is associated with a unimodal distribution (panel A2). In the panels corresponding to row B the kinetic rates of layer I were amplified by a factor of 10. In this case, the fluctuation levels diminish considerably for both mechanisms. The opposite occurs when the TF binding/unbinding rates decrease (panels of row C). For slow binding/unbinding rates our model predicts that the level of fluctuation increases and the histogram associated with the RM case becomes bimodal. In panels corresponding to row D, the kinetic rates of layer II were amplified by a factor of 10, which does not have much influence over the histograms. Nevertheless, slower rates in this layer promote a higher level of fluctuation, as shown in the panels of row E, in a similar way to slow rates in layer I depicted in the panels of row C. In the panels corresponding to row F, the kinetic rates of layer III were amplified by a factor of 10. At high production and degradation rates, the fluctuation level of the system produces mRNAs in a burst fashion for both mechanisms, which are not very distinguishable in this regime. Contrariwise, when the kinetic rates of this layer decrease by the same factor, the histograms are narrow and unimodal in both cases. We have also noted that the scaling of layer III has opposite consequences to the scaling of layers I and II. The time series and histograms were obtained at 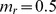. Nevertheless, the panels in column 3 show that differences between the two cooperative mechanisms are in general greater at low 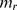 (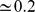) and disappear when 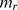 reaches one.

**Figure 9:**
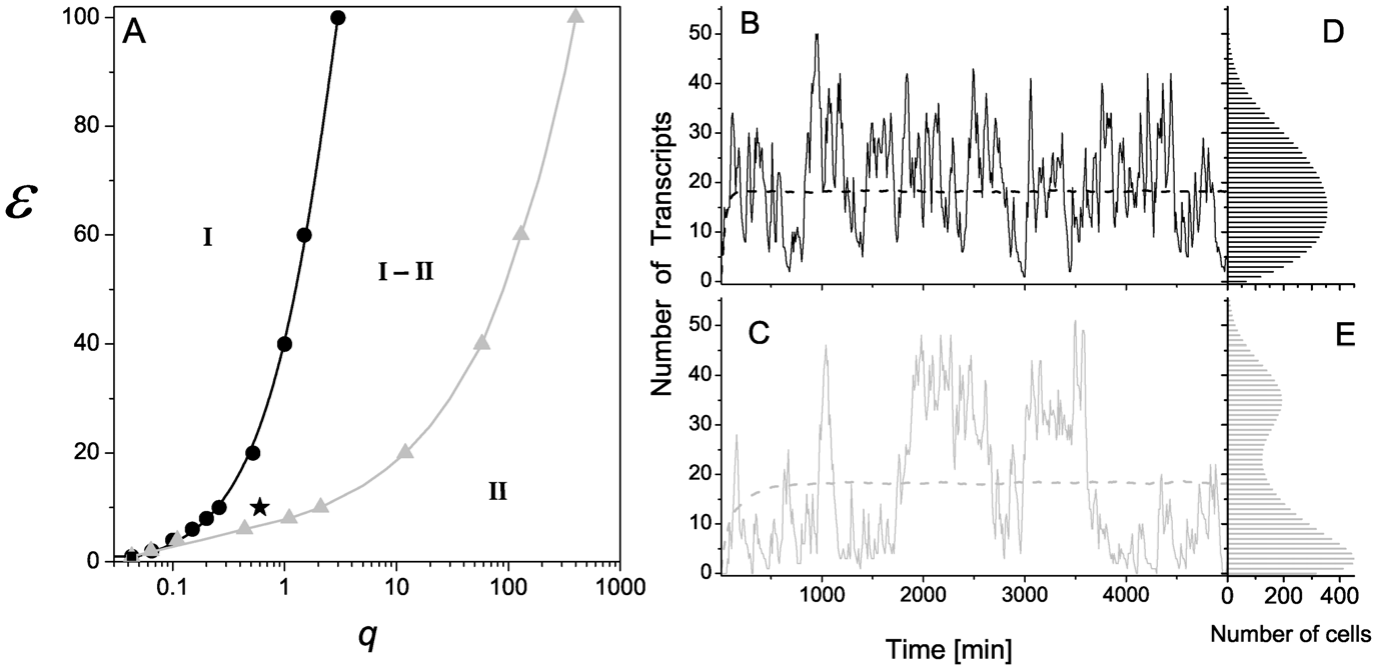
Unimodal-bimodal regimes. (A): Phase diagram in the 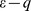 space, region I corresponds to a bimodal histogram phase, region II corresponds to a unimodal histogram phase. The black curve corresponds to the unimodal-bimodal interface of the recruitment mechanism. The gray curve corresponds to the interface of the stabilization mechanism. Symbols are obtained by simulation and lines correspond to spline fitting. Region I-II denotes an area where the histograms associated with the RM are unimodal and those associated with the SM are bimodal. (B and C) Single cell (solid line) and population average (dashed line) of the number of transcript time courses obtained by simulations using RM (B) and SM (C). (D and E) Associated histograms computed for 10000 cells. Parameters are the same as in Fig. 2A, except for 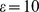, 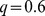 and 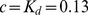, denoted by a star in panel A.

Figure 8 illustrates that both binding cooperative mechanisms are able to produce both unimodal and bimodal distributions depending on the kinetic parameters. This feature depends on the relationship between kinetic rates in layers I and III. In fact, bimodal behavior appears when the kinetics of layer I is slower than the kinetics of layer III. This was also observed in simpler models [3]. For a given kinetic relationship between these layers, there exists a region in the parameter space 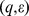 related to bimodal distribution. Parameter 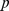 does not affect this distribution feature (data not shown). Figure 9 shows the bimodal regime for both mechanisms is in region I (low 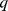), while the unimodal regime is in region II (high 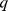). The region denoted by I-II corresponds to a region in which the unimodal regime of the RM and the bimodal regime of the SM coexist. Interestingly, the interface between these two regions depends on the acting cooperative binding mechanism and when the RM is acting, lower values of 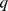 are required to get a unimodal distribution. In Figs. 9B–9E we can see the dynamic behavior and histogram for 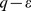 values indicated by a star in the phase diagram (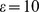, 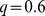, 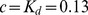 and other values are the same as in Table 1 for the activator). Thus, bimodality can also be a consequence of the cooperative binding mechanism. But at sufficiently lower unbinding rates 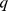, cooperative binding is not necessary to reach bimodal response.

## Discussion

Despite the rich variety of gene regulatory mechanisms acting at the transcriptional level [16], [17], [20], [28], most models consider only one or two states for the CRS. These models approximate the transcriptional control by using a regulatory expression function (Hill function in [11], [22], [37], [38] or an *ad-hoc* function to fit the model to the experimental data in [29], [30]).

We have shown that cooperative regulatory function can be derived from a model based on the law of mass action for elementary reactions [27], which allows understanding the consequences of TF cooperative interactions from first principles. For example, we have shown that response steepness depends on the energy involved in the interaction between TFs. However, this analysis was restricted to activator switches. Consequently, in this study we have generalized our previous approach in order to model repressor and biphasic switches. In our model, the basic components of the CRS can be arranged in different ways to modulate gene expression in response to a given signal. For example, a repressor molecule bound to DNA can block further assembly by interacting with general factors of the transcriptional complex [28], [39]. This aspect can be modeled in our approach, representing a repressor switch with cooperative response. Many features of the response associated with this switch are analogous to others reported previously for cooperative activator switches [27]. For example, as expected, the Hill function with integer exponent is recovered for infinity interaction energy [40], [41]. Furthermore, we show that, for switches such as the one depicted in Fig. 1, fluctuation levels associated with the recruitment cooperative binding mechanism never exceed those associated with the stabilization mechanism.

We also show two types of switches related to a biphasic response, namely, the CRS that allows full activation when the regulatory TF occurs within a narrow concentration band. The biphasic response has been reported underlying a variety of mechanisms [42]–[44]. For example, Kruppel in Drosophila acts as an activator at low levels but dimerizes at high concentrations and acts as a repressor in the same binding site [45]. Recently, it was observed that E3f1 had a biphasic response to MYC [44]. Yet another mechanism known as transcriptional interference [46] was reported to respond biphasically [47]. Our model illustrates that biphasic responses can also arise from two other mechanisms: (i) when an intermediate occupancy number of binding sites promotes the formation of the transcriptional complex, while inhibition occurs at low and high binding site occupancy numbers; (ii) when the transcriptional complex has a poor ability for RNAPol recruitment or activation and a consequent low rate of mRNA synthesis at low and high occupancy numbers. The former mechanism appears to be associated with a lower fluctuation level than the latter.

It is commonly accepted that systems that present bistability (i.e., two stable steady states under the same external conditions) are associated with a bimodal response. In this sense, some mathematical models provide examples for that [23], [48]. However, Walcsak et al. showed that an open regulatory cascade with sufficiently strong regulation can also constitute a mechanism for bimodality [49]. More recently and from a perspective of population balance, it has been shown that bistability is neither sufficient nor necessary for bimodal distributions in a population [50].

On the other hand, the all-or-none phenomenon has been observed in inducible gene expression and has been attributed to a purely stochastic origin. Several stochastic models of gene expression suggest that fluctuations in the binding/unbinding of TFs to/from DNA can explain both graded and binary responses to inducing stimuli [3], [51]–[54]. Pirone and Elston showed that the slow transitions are responsible for binary responses, whereas fast transitions produce graded responses [51]. Even though their model contemplates several regulatory binding sites, they do not consider the effects of cooperative binding on the inducible response. In the context of cooperativity, Sanchez et al. developed a repressor model that includes two regulatory sites [55]. In their model, cooperativity acts by decreasing the unbinding rate and is equivalent to our SM case. These authors found that induced responses change from long-tailed to bimodal distribution when the cooperative factor increases (see Fig. 3C in [55]). When SM is acting, simulation results from our model are in agreement with this previous observation which could be expected because, in this case, cooperativity is slowing CRS transitions. Notably, our model suggests that bimodal distributions are also promoted by the cooperative RM when cooperativity is reflected in binding rate increases, which in turn accelerate CRS transitions. To our knowledge, this has not been previously reported and adds new insight to the origin of bimodality and the effects of cooperative binding on gene expression. In particular, our finding that cooperative binding promotes bimodal distributions could explain the bimodal response observed in a stably integrated NF-AT construct in clones of the Jurkat T-cell line [56]. NF-AT molecules bind cooperatively to DNA as has been reported in [57], [58] and the construct employs three tandem copies of the NF-AT-binding site. The phase diagram obtained for our model shows that bimodal distribution can be obtained for high interaction energy between TFs even for high unbinding rates in SM. The unimodal and bimodal phases in the 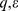-space are delimited by a cooperative binding mechanism dependent curve. Thus, there is a region in the space parameter 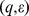 where SM shows a bimodal response while RM is associated with an unimodal regime.

Summarizing, our results with regard to the stochastic model for gene expression suggest that the gene expression regulatory architecture is measurably reflected in its associated mean response and intrinsic noise profiles.
